# Glucocorticoid Therapy in COVID-19-Induced Organizing Pneumonia: A Rare Occurrence

**DOI:** 10.7759/cureus.33991

**Published:** 2023-01-20

**Authors:** Fereshteh Yazdi, Uzoamaka Ogbonnah, Cesar Davila-Chapa, Prathik Krishnan

**Affiliations:** 1 Internal Medicine, Louisiana State University (LSU) Health Shreveport, Shreveport, USA; 2 Pulmonary and Critical Care Medicine, Louisiana State University (LSU) Health Shreveport, Shreveport, USA

**Keywords:** covid-19 hospitalization, covid-19 mortality, glucocorticoid therapy, covid-19 infection, organizing pneumonia

## Abstract

Background

Although the incidence of post-COVID-19 organizing pneumonia (OP) is low, the mortality and morbidity in select patients appear to be high. Anticipating specific populations who may be at higher risk and initiating treatment earlier could reduce mortality.

Research question

Does treatment with high dose, standard dose, or no glucocorticoids for COVID-19 infection impact the incidence and clinical outcome in COVID-19-induced OP?

Study design and methods

This was a single-center, retrospective, observational cohort study conducted from 03/01/2020 to 06/30/2021 in hospitalized patients over the age of 18 with confirmed COVID-19 infection and computed tomography (CT) scan evidence of OP. Institutional review board (IRB) approval was obtained from the institution (STUDY00002241). Patients’ demographics and oxygen requirements at the time of diagnosis, at the time of discharge, and at one, three, six, 10, and 12 months post-discharge were obtained. The dose, duration, and choice of glucocorticoid therapy were recorded for each subject, as well as oxygen requirements during hospitalization. Despite radiological evidence of OP, patients on minimal supplemental oxygen requirements did not receive high-dose or long-duration glucocorticoid therapy.

Results

A total of 881 patients were admitted with COVID-19, of which 42 met the study criteria. Three patients underwent a lung biopsy to confirm the diagnosis of organizing pneumonia. All other patients were diagnosed based on CT imaging and clinical presentation. Of the patients, 17% did not receive any steroid treatment, while 36% received dexamethasone and 43% received prednisone. The most common oxygen requirement at the time of discharge for steroid-treated patients was nasal cannula (55%) and room air (29%). The incidence of OP in this patient population was 0.05 with a mortality rate of 14%.

Interpretation and relevance

The incidence of post-COVID-19 OP appears to be lower than anticipated. Steroids for patients on lower supplemental oxygen requirements were discontinued although they had radiological evidence of OP. Patients who were on higher supplemental oxygen requirements at 10 days were continued on steroids regardless of imaging. The decision to continue steroids should be based on individual patient characteristics such as oxygen requirements. In the future, larger multicenter cohort studies would help understand further treatment of post-COVID-19-associated OP. Anticipating specific populations who may be at higher risk and starting treatment earlier could help reduce mortality.

## Introduction

Organizing pneumonia (OP) and its variant, acute fibrinous and organizing pneumonia, are well-known complications of severe viral infections. Recent reviews of postmortem biopsies and computed tomography (CT) imaging in patients suggest that a subset of patients develop secondary organizing pneumonia (OP) following infection with SARS-CoV-2.

OP is a type of interstitial lung disease (ILD) that has been associated with various known etiologies, such as infections (especially viral), drugs, rheumatologic pathologies, aspiration, or radiation therapy [1]. Clinical symptoms of secondary OP are usually described as nonspecific flu-like symptoms that persist over several weeks [1] after an initial insult. Radiological findings of OP on CT imaging predominantly show evidence of peripheral consolidations, ground glass opacities, bronchiectasis, pleural effusion, and thickening of the adjacent pleura [2-4].

Although the full extent of long-term pulmonary complications from COVID-19 remains unknown, given the number of infections, even a small proportion of patients with a persistent functional deficit from secondary OP represents a significant disease burden [3]. Given the functional limitations of secondary OP and the long duration of symptoms, it is imperative for studies to follow eligible study subjects for several months after diagnosis to uncover the most clinical value. To this effect, we conducted a retrospective study of patients with persistent hypoxia following COVID-19 infection for over 12 months. The goal of our study was to identify whether using high-dose steroids in COVID-19-infected patients with persistent symptoms and radiological features of OP has better clinical outcomes compared to those with standard dexamethasone therapy versus no steroid therapy at all.

## Materials and methods

Study design

We conducted a single-center, retrospective, observational cohort study of hospitalized patients with COVID-19 pneumonia confirmed by polymerase chain reaction (PCR) assay testing between 03/01/2020 and 06/30/2021. The intended subjects were those patients who survived initial pneumonia but were found to have persistent respiratory symptoms with radiological evidence of organizing pneumonia (Table 1). Eligible study subjects were then divided into those who were treated with high-dose prednisone (60-80 mg), standardized COVID-19 treatment dexamethasone dose (6 mg), combination therapy, or no steroids at all. Institutional review board (IRB) approval was obtained prior to initiating the study.

**Table 1:** Inclusion and exclusion criteria *Patients with alternative explanations for their respiratory symptoms (chronic respiratory disease, recurrent infection, ILD, etc.) were excluded from the study. **Patients with histological evidence of OP were included in the study (n=3). PCR: polymerase chain reaction, COVID-19: coronavirus disease 2019, CT: computed tomography, OP: organizing pneumonia

Inclusion criteria	Exclusion criteria
Positive PCR COVID-19 infection requiring hospitalization	Age < 18 years
Persistent respiratory symptoms post-COVID-19 infection post-discharge*	Persistent respiratory symptoms explained by an alternative diagnosis
CT scan with evidence of OP**	CT imaging consistent with an alternative diagnosis

Patient selection

All study participants were selected from Louisiana State University Health Hospital in Shreveport, LA, USA. Each subject had to be 18 years of age or older and had a record of positive PCR COVID-19 infection and associated hospitalization from respiratory failure. Those who had returned to the hospital or outpatient clinic with continued hypoxia with radiological studies (CT scan) suggestive of organizing pneumonia were selected for further analysis (Table 1).

Data collection and clinical assessment

All data were extracted from the health system’s medical record system, Epic. The data extracted included the following: demographic information (age, sex, and patient-reported race and ethnic group), body mass index (BMI) recorded within the duration of our study, and chronic medical comorbidities (chronic obstructive pulmonary disease (COPD) or emphysema, diabetes mellitus, obesity, hypertension, hyperlipidemia, or cancer). Subjects were assessed based on initial oxygen requirements at the time of COVID-19 diagnosis and oxygen requirements at the time of discharge, as well as one, three, six, 10, and 12 months post-discharge. Glucocorticoid therapy, dose, and duration were collected when applicable, and oxygen requirements at the time of steroid therapy initiation were compiled. Patients in both critical care settings and general medicine units were included in this study. Patient records were then followed to isolate those with persistent hypoxia and radiological evidence of organizing pneumonia to determine the overall outcome of steroid therapy during the initial COVID-19 infection.

## Results

Characteristics of COVID-19-positive patients

We identified a total of 881 patients admitted with COVID-19, of which 42 met the study criteria of having COVID-19-associated organizing pneumonia. Age ranged from 18 to 73 years with a median age of 60.5 years and a mean of 53.4 years. Of the study subjects, 52% were male. The average BMI for the subject population was 33.6 kg/m^2^. More than 50% of the study subjects had at least 1-3 comorbidities at the time of infection. Out of the 42 subjects, 13 were intubated and mechanically ventilated at the time of admission to the hospital. The most common mode of oxygen delivery to the remainder of the subjects at the time of admission was via nasal cannula. At the time of discharge, three patients remained mechanically ventilated, while 23 were discharged home with oxygen via nasal cannula. Twelve subjects left the hospital without further oxygen requirements (Table 2).

**Table 2:** Summary of patient characteristics, oxygen requirements, and glucocorticoid therapy *COPD or emphysema, diabetes mellitus, obesity, hypertension, hyperlipidemia, or cancer COPD: chronic obstructive pulmonary disease, BMI: body mass index, BiPAP: bilevel positive airway pressure

Characteristic		Value
Age in years, average		53.43
Gender, number (%)		
	Female	20 (48%)
	Male	22 (52%)
BMI in kg/m^2^, average		33.61
Race, number (%)		
	Black	23 (55%)
	Hispanic	3 (7%)
	Native American	1 (2%)
	White	1 (2%)
	Unknown	14 (33%)
Comorbidities*, number (%)		
	0	6 (14%)
	1	12 (29%)
	2	7 (17%)
	3	10 (24%)
	4	6 (14%)
	5	1 (2%)
Mechanically ventilated on admission, number (%)		
	No	29 (69%)
	Yes	13 (31%)
Oxygen requirement at diagnosis, number (%)		
	BiPAP	9 (21%)
	High-flow nasal cannula	11 (26%)
	Nasal cannula	11 (26%)
	Room air	3 (7%)
	Ventilator	8 (19%)
Oxygen requirement at discharge, number (%)		
	BiPAP	1 (2%)
	Deceased	3 (7%)
	Nasal cannula	23 (55%)
	Room air	12 (29%)
	Ventilator	3 (7%)
Glucocorticoids, number (%)		
	None	7 (17%)
	Dexamethasone 6 mg	15 (36%)
	Dexamethasone and prednisone	1 (2%)
	Hydrocortisone and prednisone	1 (2%)
	Prednisone 60-80 mg	18 (43%)
Duration of therapy, days		15.6
Deceased, number (%)		6 (14%)

Glucocorticoid therapy

Out of the 42 patients included in the study, 35 were treated with glucocorticoids during hospitalization. Eighteen patients were treated with high-dose prednisone (60-80 mg), while 15 were treated with dexamethasone (6 mg) for routine COVID-19 treatment. One subject was treated with high-dose prednisone after completing the 10-day dexamethasone therapy, while one was treated with hydrocortisone prior to transitioning to prednisone. Seven patients were not treated with any glucocorticoids at all during hospitalization. The average duration of therapy in this study was 15.6 days. Out of the patients treated with high-dose prednisone, four were deceased, and one was lost to follow-up. There were no reported deaths in the dexamethasone group. One patient with no steroid treatment was deceased in our study population.

Oxygen requirements

Out of the patients treated with high-dose prednisone, the average oxygen requirement at discharge was 3.5 L via nasal cannula. Most patients had no oxygen requirements at one-month follow-up, with no subjects requiring supplemental oxygen at 12 months follow-up. In the dexamethasone group, the average oxygen requirement at discharge was 2 L via nasal cannula, with only one patient persistently requiring 2 L of oxygen at 12 months follow-up. In the no glucocorticoid group, most patients were discharged with no supplemental oxygen, and there were no subjects who required oxygen at 12 months follow-up.

Organizing pneumonia diagnosis

Three patients underwent a lung biopsy to confirm the diagnosis of organizing pneumonia. All other patients were diagnosed based on CT imaging and clinical presentation.

## Discussion

This study examined the correlation between steroid therapy during COVID-19 infection and the development of secondary organizing pneumonia. It was found that the incidence of post-COVID-19 OP appears to be lower than anticipated, with an incidence rate of roughly 0.05 in our study population. A significant proportion of patients had at least 1-3 underlying medical conditions. The determining factor for the continuation of glucocorticoid therapy in our study population was the patient’s clinical presentation and oxygen requirements, irrespective of radiological evidence of OP on chest imaging. Most patients with low supplemental oxygen requirements had good clinical outcomes, with most patients requiring no further oxygen at 12 months follow-up. Patients who were on higher supplemental oxygen requirements after 10 days of steroid therapy (>2 L nasal cannula) were continued on steroid treatment, and the mortality rate was at approximately 14%. We conclude that the incidence of post-COVID-19-associated OP is low in this small cohort of patients, and the decision to continue steroids should be individualized to the patient and their respiratory requirements.

There have been previous reports of secondary OP associated with contagious respiratory viruses responsible for previous pandemics, such as severe acute respiratory syndrome (SARS), Middle Eastern respiratory syndrome (MERS), and influenza A [5,6]. Although histological assessment is required to confirm the diagnosis, a biopsy is not routinely performed due to the severity of the patient’s condition. The main management strategy for OP is the use of high-dose corticosteroids (methylprednisolone 500-1,000 mg for 3-5 days, followed by 0.75-1 mg/kg prednisone), which is significantly higher than the dose used for the treatment of COVID-19 pneumonia (6 mg of dexamethasone daily for up to 10 days) [3,7]. In prior studies, it has been shown that patients with persistent radiological and clinical features of OP post-COVID-19 infection benefitted from resuming oral glucocorticoid therapy with resolution of their symptoms in an outpatient setting [3]. Consistent with the current literature, we redemonstrate clinical improvement in patients with COVID-19-associated OP. However, in our study, glucocorticoid therapy was not initiated exclusively in those with radiological evidence of OP. Also, steroid therapy in our patient population was at a lower dose than routinely used for OP treatment. Continuous glucocorticoid therapy in our patient population correlated with clinical evidence of OP more so than radiological evidence. We demonstrate the importance of individualizing therapy based on the patient’s pulmonary functional status post-COVID-19 infection.

Our study limitations stem mainly from the small sample size. Another limitation of the study is relying on CT imaging for the diagnosis of OP, as histological evidence of OP was only available in three of the subjects studied. This study is also single-centered, and the prevalence of OP post-COVID-19 cannot be generalized to other populations based on our findings. Although the incidence of post-COVID-19 OP is low, the mortality and morbidity in select patients appear to be high. Anticipating specific populations who may be at higher risk and starting treatment earlier could help reduce mortality. Larger cohort studies are needed to help develop better treatment strategies.

In the future, larger multicenter cohort studies would help understand further treatment of post-COVID-19-associated organizing pneumonia.

## Conclusions

The incidence of post-COVID-19 OP appears to be low. In this study, patients treated with high-dose prednisone (60-80 mg) versus dexamethasone 6 mg versus the combination of the two treatments were evaluated and followed for one year post-COVID-19 infection. In our study, steroid treatment in patients with lower supplemental oxygen requirements was discontinued despite radiological evidence of OP. Patients who were on higher supplemental oxygen requirements at 10 days were continued on steroids regardless of imaging. The decision to continue steroids should be based on individual patient characteristics, and no clinical outcome difference was noted 12 months post-discharge between patients treated with high-dose prednisone versus dexamethasone 6 mg.
